# Novel Patient Metastatic Pleural Effusion-Derived Xenograft Model of Renal Medullary Carcinoma Demonstrates Therapeutic Efficacy of Sunitinib

**DOI:** 10.3389/fonc.2021.648097

**Published:** 2021-03-26

**Authors:** Alex Q. Lee, Masami Ijiri, Ryan Rodriguez, Regina Gandour-Edwards, Joyce Lee, Clifford G. Tepper, Yueju Li, Laurel Beckett, Kit Lam, Neal Goodwin, Noriko Satake

**Affiliations:** ^1^ Department of Pediatrics, UC Davis School of Medicine, Sacramento, CA, United States; ^2^ The Jackson Laboratory, Sacramento, CA, United States; ^3^ Department of Pathology & Laboratory Medicine, UC Davis School of Medicine, Sacramento, CA, United States; ^4^ Department of Biochemistry and Molecular Medicine, UC Davis School of Medicine, Sacramento, CA, United States; ^5^ Genomics Shared Resource, UC Davis Comprehensive Cancer Center, Sacramento, CA, United States; ^6^ Department of Public Health Sciences, UC Davis, Davis, CA, United States

**Keywords:** renal medullary carcinoma, sunitinib, multikinase inhibitor, patient-derived xenograft model, metastatic pleural effusion

## Abstract

**Background:**

Renal medullary carcinoma (RMC) is a rare but aggressive tumor often complicated by early lung metastasis with few treatment options and very poor outcomes. There are currently no verified RMC patient-derived xenograft (PDX) mouse models established from metastatic pleural effusion (PE) available to study RMC and evaluate new therapeutic options.

**Methods:**

Renal tumor tissue and malignant PE cells from an RMC patient were successfully engrafted into 20 NOD.Cg-Prkdc^scid^ Il2rg^tm1Wjl^/SzJ (NSG) mice. We evaluated the histopathological similarity of the renal tumor and PE PDXs with the original patient renal tumor and PE, respectively. We then evaluated the molecular integrity of the renal tumor PDXs between passages, as well as the PE PDX compared to two generations of renal tumor PDXs, by microarray analysis. The therapeutic efficacy of sunitinib and temsirolimus was tested in a serially-transplanted generation of 27 PE PDX mice.

**Results:**

The pathologic characteristics of the patient renal tumor and patient PE were retained in the PDXs. Gene expression profiling revealed high concordance between the two generations of renal tumor PDXs (RMC-P0 vs. RMC-P1, r=0.865), as well as between the first generation PE PDX and each generation of the renal tumor PDX (PE-P0 vs. RMC-P0, r=0.919 and PE-P0 vs. RMC-P1, r=0.843). A low number (626) of differentially-expressed genes (DEGs) was seen between the first generation PE PDX and the first generation renal tumor PDX. In the PE-P1 xenograft, sunitinib significantly reduced tumor growth (p<0.001) and prolonged survival (p=0.004) compared to the vehicle control.

**Conclusions:**

A metastatic PE-derived RMC PDX model was established and shown to maintain histologic features of the patient cancer. Molecular integrity of the PDX models was well maintained between renal tumor and PE PDX as well as between two successive renal tumor PDX generations. Using the PE PDX model, sunitinib demonstrated therapeutic efficacy for RMC. This model can serve as a foundation for future mechanistic and therapeutic studies for primary and metastatic RMC.

## Introduction

Renal medullary carcinoma (RMC) is a rare but remarkably aggressive tumor that predominantly affects young black males with sickle cell trait. Since its first description by Davis et al. in 1995 (1), fewer than 230 cases have been reported in the literature (2–4). RMC is characterized by high rates of metastasis at diagnosis and resistance to both chemotherapy and radiation (5, 6), contributing to a median time from diagnosis to death of approximately 8 months (2). Lung metastases causing malignant pleural effusion (PE) are especially common and devastating (4). The current standard-of-care treatment for RMC is radical nephrectomy with retroperitoneal lymph node dissection. This is followed by systemic chemotherapy (7) to address the widespread metastasis. Although platinum-based cytotoxic regimens for RMC have been tested in some cases, the maximum response rate was only 29% (8). In light of the absence of effective treatment options for RMC, clinical trials have formed the core of recommended treatment. However, it is difficult to study RMC due to the extreme rarity of the cases and existence of very few disease models, including cell line or *in vivo* models. In particular, there are no *in vivo* models for RMC created using metastatic PE.

Patient-derived xenograft (PDX) models, established by engrafting patient tumor cells into immunodeficient mice in order to replicate the cancer, have become a common tool to study new anticancer agents. PDX models are advantageous, compared with cell lines or murine disease models, due to their ability to recapitulate the molecular and cellular traits, disease progression, and chemotherapeutic response patterns of the original patient tumor. These models can then be maintained by successive passaging through generations of xenograft mice, resulting in an easily generated and consistent platform for preclinical and mechanistic studies. To effectively evaluate novel anticancer agents to treat RMC, an established PDX model is first needed. A metastatic site-derived PDX model is ideal for a cancer like RMC to develop effective treatments to address drug resistance. However, there have not been any histopathologically and molecularly verified RMC PDX models of any sites of metastasis to date.

In this study, we established an RMC PDX model generated from malignant PE that corresponds histologically to the patient cancer. This PE model is then applied toward demonstration of the anticancer agent sunitinib as a viable treatment option for RMC.

## Methods

### Mouse Xenograft Model

Tumor tissue and malignant PE cells were obtained from the patient at nephrectomy and thoracentesis, respectively, using the UC Davis Internal Review Board approved protocol. Tissues and cells were transferred upon collection to The Jackson Laboratory (Sacramento, CA) where all animal experiments were performed, in accordance with the Animal Care and Use Committee of the Jackson Laboratory and conformed to the recommendations in the National Academy of Sciences Guide for the Care and Use of Laboratory Animals (9). The tissue was dissected into small fragments (approximately 3 mm^3^) and implanted subcutaneously to the flank of 10 healthy 6 to 8-week-old NOD.Cg-Prkdc^scid^ Il2rg^tm1Wjl^/SzJ (NSG) female mice (The Jackson Laboratory) using a 10-gauge trocar. Tumor cells were isolated from the PE by centrifugation, then inoculated subcutaneously (1.0 × 10^7^ cells per injection) to the flank of 10 additional NSG mice (PE-P0). Once the PE-P0 tumor size reached 1000 mm^3^, the tumors were harvested, fragmented into 3 to 4 mm^3^ sections, and transplanted into recipient mice (PE-P1) for drug efficacy studies. Tissue from the patient primary tumor, patient PE, renal tumor xenograft, and PE-P0 xenograft were submitted to the UC Davis Department of Pathology and Laboratory Medicine for standard histologic processing, embedding, and H&E staining.

### Drug Efficacy Studies

Sunitinib malate (Selleckchem) and temsirolimus (Selleckchem) were compared to a vehicle control for tumor growth delay and overall survival in PE-P1 mice. 27 PE tumor bearing mice were enrolled into one of three treatment groups when individual tumor volumes reached ~300 mm^3^. Group 1: vehicle control with 0.5% carboxymethylcellulose (CMC) and dextrose 5% in water (D5W) (n = 10); Group 2: sunitinib malate (n = 10); Group 3: temsirolimus (n = 7). All animals in the control group simultaneously received both 0.5% CMC by oral gavage for 3 weeks at 5 days on, 2 days off per week and D5W by intravenous tail vein administration for 5 days. Sunitinib was dissolved in 0.5% CMC and administered by oral gavage at 50 mg/kg for 3 weeks at 5 days on, 2 days off per week. A 50 mg/ml temsirolimus stock solution was dissolved in 100% ethanol and diluted to 2 mg/ml in D5W prior to dosing by intravenous tail vein administration at 20 mg/kg for 5 days. Tumors were measured 3 times a week for 94 days after treatment initiation or until tumor volumes reached 2000 mm^3^.

### Tumor Growth Analysis

Tumor volumes (V) were calculated from digital caliper raw data using the formula V (mm^3^) = (l x w^2^)/2. The value *w *(width)* *was the smaller of two perpendicular tumor axes and the value *l* (length) was the larger of two perpendicular axes. The tumor endpoint volume was set at 2000 mm^3^ and tumors were monitored until the animals were euthanized either due to tumors reaching 2000 mm^3^ or animals reaching the study endpoint at 94 days post drug treatment initiation. Tumor doubling times were calculated by fitting mixed-effects growth-curve models to log-transformed volumes separately for each treatment group, transforming the estimated group-specific slope and its 95% confidence interval (CI) to percent change, and solving for the time to double in volume and the corresponding time to reach 1000 mm^3^ (10). This approach made use of all available volume measurements even in animals that did not reach 2000 mm^3^.

Mean tumor volume at each day of measurement with standard error were calculated and plotted for each treatment group. Animals that had necrotic or ulcerated tumors requiring euthanasia prior to their tumors reaching 2000 mm^3^ were excluded from statistical analysis. Statistical significance for testing equality of treatment group distributions of time (days) to reach tumor size 2000 mm^3^ values for treatment group comparisons was determined by the log-rank test. A 95% confidence value was used for two-tailed statistical analyses. Kaplan-Meier survival curves were plotted to illustrate the time to reach tumor size 2000 mm^3^ for the three groups. Animals that did not reach the 2000 mm^3^ were censored at 94 days. Analyses used either Prism 8.3 software (GraphPad) or SAS Version 9.4. Results were reviewed by two statisticians independently.

### Gene Expression Profiling

Total RNA was isolated from the PE xenograft tumor and two generations of the renal tumor xenograft tumor. Comprehensive gene expression profiling was performed using Human Gene 1.0 ST microarrays (Affymetrix) which enable analysis of 28,869 well-annotated genes (i.e., based on the March 2006 human genome sequence assembly, NCBI build 36, UCSC Hg18). GeneSpring GX 11.5 software (Agilent Technologies) was utilized for data analysis. The ExonRMA16 algorithm was applied for probe summarization, followed by quantile normalization and baseline transformation of probe intensity values from the raw data CEL files. Statistical testing for differential gene expression (i.e., PE-P0 vs. RMC-P0; moderated T-test, Benjamini-Hochberg FDR-adjusted p<0.05) was performed and followed by hierarchical clustering and heatmap visualization of the results.

## Results

### Case

**Figure 1:**
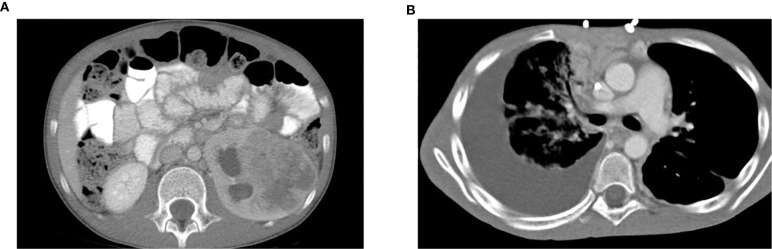
A patient presents with a large left renal mass and a subsequent large right pleural effusion. **(A)** CT scan of the abdomen on initial presentation showed a left renal mass measuring 6.8 x 5.1 x 7.3 cm with diffuse necrotic change. No involvement of the right kidney was identified. **(B)** CT scan of the lung six months later showed a rapidly developed large right PE. Worsening of pulmonary metastases with development of lymphangitic carcinomatosis was also noted.

**Figure 2:**
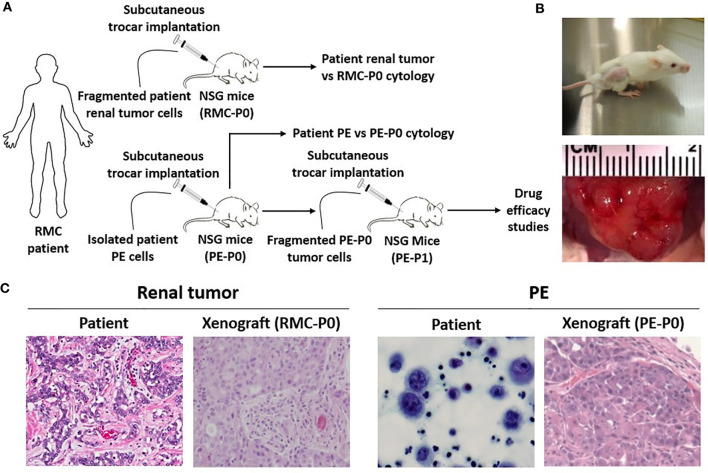
RMC patient-derived xenograft models are created using NSG mice. **(A)** Experimental scheme for subcutaneous transplantation of patient renal tumor and PE cells into the flank of NSG mice. Fragmented PE tumor cells from the P0 group were serially transplanted into the P1 group for drug efficacy studies once P0 tumor size reached 1000 mm^3^. **(B)** Tumor development in secondary mice. Tumor size can be seen in the flank of the mouse (top) and through gross observation of the tumor during necropsy (bottom). **(C)** Classic histopathologic features of RMC were noted in the patient renal tumor, including irregular glands and nests of epithelial cells in a vascular, desmoplastic stroma. These features were replicated in the xenograft. Both patient PE cytology and PE-derived xenograft pathology showed essentially undifferentiated malignant cells.

The patient was a 9-year-old previously healthy male with sickle cell trait who presented with intermittent gross hematuria. He was found on CT to have a large left renal mass with lymph node involvement (**Figure 1A**) and multiple liver and lung metastases (not shown). Left nephrectomy was performed. Pathologic analysis of the primary renal tumor revealed classic histopathologic features of RMC, including highly atypical epithelioid tumor cells with large vesicular nuclei and nucleoli arranged in irregularly solid nests, infiltrative peripheries, cribriform structures, and complex glandular forms (**Figure 2C**). Tumor cells were associated with abundant desmoplastic to sclerotic stroma. Immunohistochemical staining of the tissue showed 2+ VEGF, negative CD117, normal topoisomerase IIα, and loss of INI-1 expression. Karyotype analysis showed normal male phenotype, 46, XY. The patient was treated by chemotherapy, including bortezomib, paclitaxel/carboplatin/bevacizumab, doxorubicin/bevacizumab, and sunitinib (**Supplemental Table 1**). Treatment regimens were chosen based on the patient’s tumor pathology, previously reported regimens of RMC (6, 11–17), and internal tumor board discussions. Further details and rationale for each regimen are summarized in **Supplemental Table 1**. The patient showed partial or no response to each regimen, and six months after diagnosis, developed a large PE (**Figure 1B**). Cytologic analysis of the PE cells showed large, highly pleomorphic malignant cells with hyperchromatic nuclei admixed with inflammatory cells and reactive mesothelial cells (**Figure 2C**). The PE was drained and the patient received several more courses of chemotherapy; however, the cancer continued to progress, and he died nine months after diagnosis.

### PDX Models Are Successfully Created Using Patient Renal Tumor and Malignant PE Cells and NSG Mice

**Figure 3:**
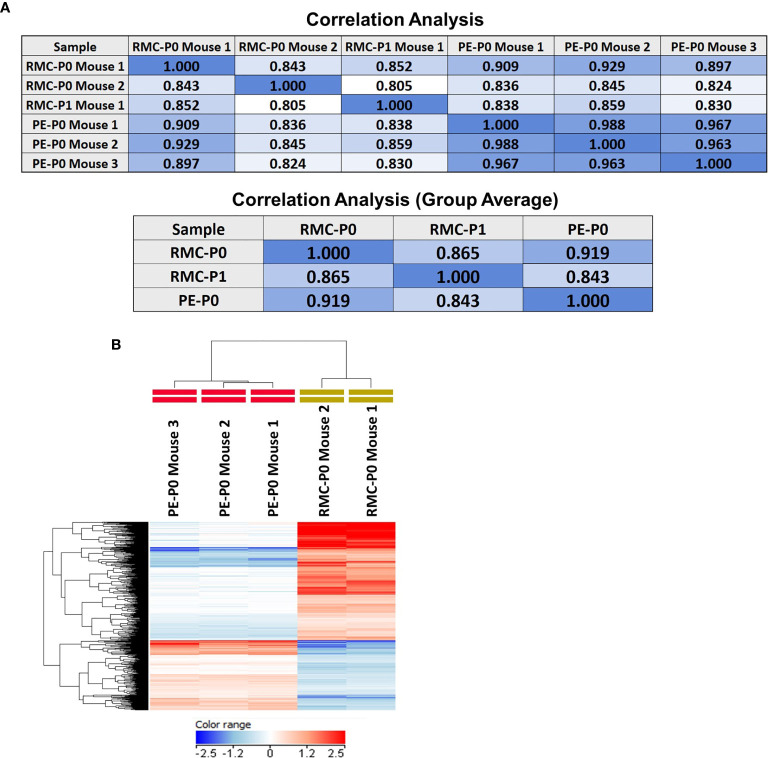
Gene expression profiling demonstrates that the PE model faithfully recapitulates the molecular features of the renal tumor PDX. Microarray gene expression profiling of the P0 and P1 renal tumor PDX and the PE PDX tumors was performed as described in *Methods*. **(A)** Pearson correlation analysis performed on the filtered expression data demonstrated high concordance between the PE PDX and each of the renal tumor PDXs, as well as between both generations of renal tumor PDX. There was also very high concordance between individual PE PDX samples. Coefficients are presented in correlation matrices. **(B)** Differential expression analysis was performed by group-wise comparison of PE-P0 vs RMC-P0 PDX data (moderated T-test, FDR-corrected p<0.05) followed by hierarchical clustering of the DEGs. The results were visualized with a heat map and demonstrate that the RMC-P0 and PE-P0 samples cluster as two distinct branches of the dendrogram based on clustering of their gene expression patterns depicted by relatively higher (red) or lower (blue) expression across the individual samples and tumor types.

Direct subcutaneous flank implantation of both fragmented patient renal tumor tissue and isolated PE cells gave rise to large tumors in 10 renal tumor xenograft mice (RMC-P0) and 10 PE-P0, respectively (**Figures 2A, B**). Serial transplantation of PE-P0 tumor cells into PE-P1 mice also resulted in robust tumor development in all PE-P1 mice. The pathologic features of the patient renal tumor and patient PE were recapitulated in the RMC-P0 tumor and PE-P0 xenografts, respectively (**Figure 2C**). The renal tumor and PE xenografts were then evaluated for their conservation of molecular integrity by determining if they possessed similar molecular features using microarray gene expression profiling. Correlation analysis of the resultant gene expression profiles demonstrated high concordance between the renal tumor xenograft generations (RMC-P0 vs. RMC-P1, group average r=0.865), as well as between the PE-P0 xenograft and the two generations of renal tumor xenografts (PE-P0 vs. RMC-P0, group average r=0.919 and PE-P0 vs. RMC-P1, group average r=0.843) (**Figure 3A**). In particular, the highest correlations were found between P0 PDXs, not only amongst the PE samples, but also between RMC-P0 vs PE-P0. Additionally, there was a low number of statistically significant differentially-expressed genes (DEGs, n=626, FDR-corrected p<0.05) between the PE-P0 xenograft and the RMC-P0 xenograft (**Figure 3B**). In summary, the gene expression characteristics of the PE-derived xenograft were highly consistent with those of the renal tumor xenografts and similar within the PE-P0 generation.

### Sunitinib Is Highly Efficacious in Reducing Tumor Growth and Improving Survival in the PE PDX Mice

**Figure 4:**
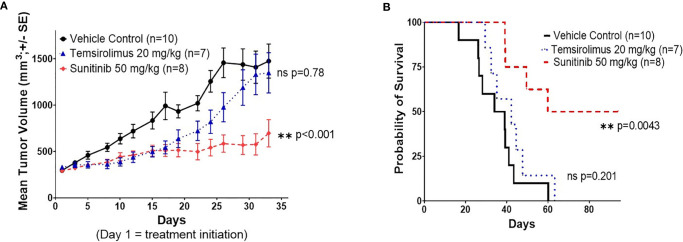
Sunitinib shows significant therapeutic efficacy in the RMC PE PDX. **(A)** Tumor volume response curve for P1 NSG mice engrafted with P0 malignant PE tumor cells treated with vehicle control, sunitinib, or temsirolimus. Treatment was initiated on day 1. Each data point represents the mean tumor size of all mice in one treatment group at a given time point, measured three times a week. Error bars represent 1 standard error from the mean. P values represent comparisons of overall growth rate calculated by fitting mixed effects models to log(volume). **(B)** Kaplan-Meier survival curve for the same study in **(A)**. Survival was measured until day 94 after xenotransplantation.

In order to determine the efficacy of sunitinib and temsirolimus as potential treatments for RMC, 27 serially-transplanted PE tumor-engrafted mice (PE-P1) were treated with these drugs. Sunitinib was selected in the setting of the patient in this case showing partial response to sunitinib monotherapy and having VEGF positive tumor staining. Temsirolimus was selected based on recent data suggesting that mTOR inhibition was effective in patients with non-clear-cell renal cell carcinoma (nccRCC) through downregulation of HIF, upstream of VEGF (18). Both drugs were also chosen based on reports of successful treatment in metastatic nccRCC (19, 20). Two out of ten mice in the sunitinib group were excluded from the study based on the study criteria described above. Sunitinib significantly attenuated tumor growth compared to vehicle control, with a tumor doubling time of 44.9 days (95% CI 38.2-54.4) vs. 14.6 (13.7 – 15.6) days (**p<0.001) (**Figure 4A**) and a time to reach tumor size of 1000 mm^3^ of 71 days vs. 22 days (**p<0.001). Sunitinib also significantly prolonged survival of the treated mice compared to vehicle control (**p=0.004) (**Figure 4B**). Temsirolimus showed a possible early attenuation of tumor growth rate but almost identical doubling time and time to reach tumor size of 1000 mm^3^ compared to vehicle control when averaged over the follow-up period (14.7 vs. 14.6 days and 29 vs. 22 days, respectively, p=0.78) and no difference in survival curves from vehicle control (p=0.201) (**Figures 4A, B**). In addition, whereas no mice survived past 60 days in the control group or 63 days in the temsirolimus group, 4 out of 8 sunitinib-treated mice remained alive, with very small tumor sizes, until study end at day 94.

## Discussion

RMC is an exceptionally rare but highly aggressive malignancy with nearly uniform fatality (21). It is generally highly resistant to chemotherapy and radiation (5, 6), with a 5-year survival rate as low as 9% (2). Early metastasis, often to the lung causing PE, is a significant contributor to mortality. Therefore, the development of effective therapies toward both the primary tumor and metastatic sites to improve outcomes is desperately needed. A disease model is necessary to develop and test new therapies. However, because of the rarity of RMC, only two mouse models, both derived from primary renal tumor tissue, have been described to date (22–24). There are no reports of RMC mouse models developed from malignant pleural fluid. In this study, for the first time, we succeeded in creating an RMC PDX model from metastatic PE cells. Furthermore, we demonstrated molecular integrity between PE xenografts and the primary renal tumor xenograft, as well as between successive PE xenograft generations. We further demonstrated the therapeutic efficacy of sunitinib in the PE xenograft model.

Since their emergence, PDX models have continued to prove their efficacy as ideal tools for the development of targeted therapies, especially in aggressive cancers (25). These models are generally established from primary tumor tissues. Additionally, several studies have discussed the ability of primary tumor PDX models to replicate patient disease metastasis and thus function as tools for investigating treatments for metastatic and resistant cancers (26). However, there have been few accounts of PDX models derived directly from patient metastases. And while PDX models established specifically from malignant PEs have primarily been reported in cancers of the lung and breast in the setting of local disease progression and spread (27, 28), very few instances of metastatic PE-derived xenografts have been described and validated in the literature (29, 30). Here, we report and validate a novel metastatic PE-derived PDX model for RMC. Whereas studies such as that by Xu et al. created PE xenograft models in part due to the relative ease of accessing patient pleural fluid samples compared to primary tumor tissue (27), few efforts have been undertaken to investigate the molecular and genetic profiles of PE versus primary tumor derived models. Our study examines those similarities through DEGs. Our model is also particularly clinically relevant as RMC usually presents with metastasis at diagnosis and is commonly complicated by rapidly developing, refractory PEs that can contribute significantly to mortality, as seen in this patient (31–33). Therefore, our PE-derived PDX model will be exceedingly useful as an experimental model due to its excellent engraftment with high similarity compared to the renal tumor and direct relevance to the course of the disease in the patient setting.

Our successful serial transplantation of renal tumor xenografts and reproducibility between passages with histopathologic similarity and high gene expression correlation demonstrates successful initial model validation. However, as the drug therapy experiments in this study were performed on the PE-derived model, it is also particularly crucial to confirm its integrity to the patient tumor, patient PE, and primary renal tumor model for the drug efficacy findings to be generalizable to patients. Solid tumor PDX models are generally verified through histopathologic and genetic analysis. A systematic review of PDX model validity by Collins et al. (34) suggests that model verification should include confirmation by an independent pathologist that the xenograft is histologically comparable to the parent tumor, genotyping to confirm that the xenograft matches the given patient from which it was established, and demonstration of serial transplantation to confirm tumor cell growth. In our study, an independent pathologist verified that the PE-xenograft replicates histopathologic features of the original patient PE. Serial transplantation of the PE model was also successfully completed. In addition, microarray analysis demonstrated high concordance of gene expression profiles between the primary tumor-derived xenografts and PE-derived xenograft with a minimal difference in DEGs. Correlation among the three tested PE PDX samples was also exceptionally high (**Figure 3A**), suggesting that the PE model is molecularly consistent. There is a low number of DEGs between the P0 renal tumor xenograft and the P0 PE xenograft. Specifically, only 626 of the 24,392 genes analyzed were differentially expressed (2.57%), demonstrating that many features of the renal tumor were conserved in the PE. The genes that were altered are likely based on the different biological properties of the PE, which was developed during the clinical course, relative to the primary site solid tumor, in addition to any alterations occurring during xenograft establishment. This suggests that our drug experiment findings in the PE xenograft model could be highly applicable to both patient metastatic and primary disease.

Drug experiments in the PE PDX model revealed the significant effect of sunitinib monotherapy on tumor growth reduction and prolonged survival. However, as monotherapy is rarely effective in treating cancers, sunitinib monotherapy in our model does not appear curative. The patient in this study also only achieved a partial response to sunitinib treatment. The report on sunitinib’s efficacy in RMC is very limited (35, 36). However, data from case reports reveal a heterogeneous response, ranging from months of stable disease to merely weeks until progression, though overall point toward the inefficacy of monotherapy and recommendation of multidrug therapy (8, 37). Altogether, sunitinib has potential as a first-line treatment option for RMC in combination with other chemotherapy drugs or specific molecular-targeted agents. To this end, our model can be used to develop a combination therapy for metastatic RMC. Temsirolimus was selected based on its reported efficacy for other non-RMC renal cancers and due to our patient tumor showing some VEGF expression (2+ by immunohistochemistry). Temsirolimus showed only modest tumor growth reduction and prolonged survival. Used as a single agent, this can be expected since survival and/or growth of our RMC might not be solely dependent on the MTOR pathway, as supported by a recent comprehensive genomic characterization study of RMC that identified no MTOR mutations in its 31-case sample (24). The study also showed that activation of c-MYC and its downstream pathway is a distinct genetic marker of RMC, further suggesting that inhibition of MTOR, which is upstream of c-MYC, is not expected to be effective. Alternatively, temsirolimus-mediated mTORC1 inhibition could result in a potent survival signal *via* phosphoinositide 3-kinase (PI3K)/AKT hyperactivation.

In future studies, drugs could be selected based on not only overexpressed genes and specific mutations detected in broader genomic studies of RMC such as by Yang et al. and Msaouel et al. (21, 24), but also patterns of DEGs identified by the gene expression profiling of this PDX model as described in this study. Furthermore, pre- and post-drug timepoint gene expression profiling and/or western blot and proteomic analysis will be useful to confirm the target genes and to understand potential disease progression and drug resistance mechanisms. This could also provide more objective markers for disease progression and treatment response, which will be clinically useful. In addition, studies to elicit understanding of the molecular pathways involved in RMC development and metastatic progression are necessary. These studies would allow for both precise targeting of affected pathways specific to this model and wide coverage of commonly mutated RMC genes more generalizable to other cases. Our PDX model can serve as a foundation for these mechanistic studies. Given the aggressive and resistant nature of RMC, these studies are critical to discovering effective therapies; specifically, multidrug therapy experiments should be explored. For example, sunitinib plus 5-methyl-cromolyn combination therapy could target both VEGF, overexpressed in this case, and S100P, shown to have 53-fold upregulation in RMC (38). The cyclic GMP-AMP synthase interferon genes (cGAS-STING) immune pathway has also recently been shown to upregulate immune checkpoints in RMC; this serves as another ideal therapeutic target for future studies (24).

One key limitation in our study is that the PDX model was based on a single patient case, largely due to the rarity of RMC. A greater diversity of cases would offer a more robust sample size that could allow our findings, especially involving therapeutic efficacy of specific drug regimens, to be applied more universally. Evidence of genomic stability between primary tumor and PDX would also be stronger with an increased case sample size, particularly as novel mutations have been described that arise in PDXs (39). However, while it is difficult to obtain more samples due to the rarity of the cancer, this model shares molecular commonalities with other reported RMC cases, increasing the external validity of this study. For example, patient pathology in this case showed *INI-1/SMARCB1* loss of expression, which is consistent with uniform loss of *SMARCB1* in several other reported cases and comprehensive studies of RMC (24, 40–42). A second limitation is the gene expression analysis of only two primary renal tumor PDX generations and one PE PDX generation. While our study showed high reproducibility between two primary tumor PDX generations for initial validation as well as high concordance between one generation of primary tumor PDX and PE PDX, analysis of additional passages of both primary and metastatic models could further confirm the integrity of the models. In particular, demonstrating high reproducibility between successive PE PDX model passages would more definitively show that it can maintain fidelity and be effectively utilized for future studies on RMC drug resistance, rescue therapy, and more. Finally, more comprehensive analysis, including DNA variant analysis and gene expression analysis for the patient tumor and PE, as well as PDX tumor and PE, could further add to the robustness of our model.

In conclusion, we successfully created a novel RMC malignant PE-derived xenograft model that recaptured the original patient tumor’s histopathologic features, as well as maintained molecular integrity between renal and PE xenografts. This model can serve as a basis for further elucidating the molecular mechanisms by which metastasis develops in RMC and for developing new treatments. It also opens the door for potentially immediate clinical application and patient benefit.

## Data Availability Statement

The datasets presented in this study can be found in online repositories. The names of the repository/repositories and accession number(s) can be found below: http://tumor.informatics.jax.org/mtbwi/pdxDetails.do?modelID=TM00387, TM00387.

## Ethics Statement

The animal study was reviewed and approved by Animal Care and Use Committee of The Jackson Laboratory. Written informed consent was obtained from the minor(s)’ legal guardian/next of kin for the publication of any potentially identifiable images or data included in this article.

## Author Contributions

JL, KL, NG, and NS contributed to the conception and design of the study. RG-E performed the histopathological analysis. RR and NG conducted the mouse experiments. YL and LB performed the statistical analysis. CT performed the analysis of the microarray gene expression profiling data. AL wrote the first draft of the manuscript. MI, CT, YL, LB, and NG wrote sections of the manuscript. All authors contributed to the article and approved the submitted version.

## Funding

This work was supported by research funding from the Keaton’s Child Cancer Alliance, The Hartwell Foundation, Hyundai Hope on Wheels, Academic Senate Research Grant, National Center for Advancing Translational Sciences, NIH, through grant UL1 TR000002, CTSC-MCRTP (NS) and St. Baldrick’s Foundation (AL). The UC Davis Comprehensive Cancer Center Genomics Shared Resource and Biostatistics Shared Resource are supported by Cancer Center Support Grant (P30CA093373) from the National Cancer Institute.

## Conflict of Interest

The authors declare that the research was conducted in the absence of any commercial or financial relationships that could be construed as a potential conflict of interest.
