# Supplementary material for: Novel Patient Metastatic Pleural Effusion-Derived Xenograft Model of Renal Medullary Carcinoma Demonstrates Therapeutic Efficacy of Sunitinib
Source: Front Oncol. 2021 Mar 26;11:648097. doi: 10.3389/fonc.2021.648097 (PMC8032976; doi:10.3389/fonc.2021.648097)
Supplement: Supplementary file 1 [file Table_1.docx]

| **Treatment Regimen** | **Cycles** | **Response** |
| --- | --- | --- |
| Bortezomib (1.2mg/m^2^)  IV twice a week for 2 weeks and 1 week off | 2 | None |
| Bevacizumab (15mg/kg) / Carboplatin (527mg/m^2^) / Paclitaxel (175mg/m^2^)  IV every 3 weeks | 2 | Partial |
| Bevacizumab (10mg/kg) / Doxorubicin (20mg/m^2^)  IV every 2 weeks | 5 | Good initially |
| Sunitinib (37.5mg/m^2^)  PO daily for 4 weeks | 2 | Partial |

**Supplemental Table 1. Patient treatment regimens and response**

All the treatment regimens were selected based on the available RMC and treatment information during the patient’s clinical course and also on tolerable regimens to maintain patient quality of life. Bortezomib was chosen as the initial treatment regimen based on a case report demonstrating a complete response of RMC to seven months of Bortezomib treatment with no evidence of disease after more than two years of follow-up (11). A phase II trial of Bortezomib for metastatic renal cell carcinoma also showed partial response (12). The patient in this study did not respond to Bortezomib, thus therapy was switched to Bevacizumab to target his tumor based on its VEGF positive immunohistochemical staining. A partial response was achieved with a bevacizumab, carboplatin, and paclitaxel combination therapy, after which a third regimen was begun using bevacizumab and doxorubicin. Response to this treatment was initially promising, however the patient’s disease eventually progressed. Sunitinib monotherapy was chosen as the subsequent treatment based on literature showing partial response in RMC (19).
